# Some Legislation That We Need

**Published:** 1892-11

**Authors:** 


### *EDITORIAL.*

The office of THE JOURNAL is in room 330 Equitable Building.
Address communications relating to the Editorial Department to DR. L. B. GRANDY, Atlanta, Ga., Box 431.
Business communications should be addressed to DR. M. B. HUTCHINS, Atlanta, Ga.
All remittances should be made payable to "ATLANTA MEDICAL AND SURGICAL JOURNAL."
Articles for publication should reach this office not later than the 15th instant.
The editors are not responsible for views expressed by contributors.

### SOME LEGISLATION THAT WE NEED.

At the season of the year when our legislators (wise and otherwise) begin] to gather themselves together it may not be amiss for us modestly to make a suggestion which we would recommend to their serious consideration. Of recent years in the legislatures of the States there has been a vast amount of misdirected energy in matters medical, which, if properly utilized, would have been productive of mutual good, both to the profession and the States. The Georgia legislature last year passed with a rush a bill prohibiting physicians from getting drunk, but strangled in committee meeting another requiring the medical colleges of the State to adopt the three-year course ; thereby assuming the Pharisaic attitude of tithing mint and anise and cummin while neglecting the weightier matters of the law.

There is one matter which the legislature of Georgia ought to consider. In fact, it can no longer afford not to do so. This State needs, and needs badly, a Board of Medical Examiners who shall pass upon the fitness or unfitness of those wishing to practice medicine within its limits. A large proportion of the citizens of any State have not intelligence enough to protect themselves from the ignorant practitioner on the one hand and the unprincipled charlatan on the other. Then it becomes the duty of the State to protect them. If those who offer their professional services to the community were everywhere what they should be, both in intelligence and integrity, there would be no cause for legislation. But the disagreeable fact remains that the medical schools of the country every year send out young men totally unqualified for practice, while there are those also so devoid of every sentiment of honor that they are willing to extract a dishonest livelihood by preying upon the ignorance and credulity of those whom they are able to deceive. Their brazen faces and lying advertisements disgrace the columns of the daily press which will print anything for money. They cure the incurable, remove the unremovable, and accomplish the impossible, all either "by consultation or by mail," "without the use of the knife," and "satisfaction guaranteed." We are glad to say that the quack's field of operation is becoming more and more restricted year by year as the light dawns upon the intelligence of State legislatures. The door of quackery is closing in this country, and those who make their shameless living out of it are being forced to seek hospitable shelter in those States yet willing to harbor them. In Georgia, which boasts of being an Empire State these Ishmaels in the profession find abundant opportunity still to ply their nefarious trade, with none to molest or make afraid.

Anybody can practice medicine in this State who has fifty cents to pay to the County Clerk. No diploma is required and no license is furnished. Georgia requires a license of her peanut venders, her pawnbrokers, her peddlers, her dairymen, her drug clerks, her insurance agents and her lawyers ; but he who proposes to treat the sick or aid the injured only registers his name in the court house in order that the sheriff may know from whom he is to collect theprofessional tax of ten dollars per year.

There are laws for the protection of game and for the prevention of cruelty to animals, from the cat to the car-mule, but the sick who do not know the standing of different physicians, whether competent or incompetent, honorable or dishonorable, have no protection.

Let us see what has been done by Examining Boards in other States. In Illinois when the Practice Act went into effect (July 1, 1887) there were 7,400 so-called physicians in the State, of whom only 3,600 were regular graduates—3,800 non-graduates. Two and one-half years operation of this act reduced the number of these non-graduates from 3,800 to 575. The total number of physicians in Illinois is less now than it was twelve years ago, in spite of increased population.

Before the Examining Board of Virginia there have appeared in the last seven years 564 applicants for license. Of these 173 or 30.6 per cent. have been rejected. Before the North Carolina Board there have been 600 applications and 178 rejections, or 29.6 per cent. The Alabama Board has rejected 13.5 per cent. of applicants for license in that State. In all the States having Examining Boards the average percentage of rejections is something over twenty-five. As to the good that may be accomplished by such a board, we have the testimony of Dr. Martin, of Virginia, who says that the law has well nigh cleared his State of frauds and humbuggery ; that it has protected unsuspecting citizens, both from the money getting quackeries of cheats and adventurers, and the injudicious, though honest efforts of unqualified doctors ; that it has stimulated the young men entering the medical profession to greater effort and higher aims; and finally that it has aroused the medical schools to better work in endeavoring to prepare their graduates to meet the requirements of the law.*

*The Mission and Methods of the Medical Examining Board of Virginia, by Dr. R. W. Martin, Chatham, Va.

Now the practical question arises, what becomes of all those medical and ethical perverts who are ejected from a tired and disgusted community, and of those twenty-five out of every hundred applicants who are every year denied the right to practice in these States? The answer is not difficult. Like other moving bodies they travel along the lines of least resistance and finally find a resting place in those places still willing to receive and patronize them. If this statement needs proof I will quote from a recent address of Dr. Draper, of Boston.*

"One State after another has passed restrictive laws of greater or less stringency with the single aim of discouraging quackery. Massachusetts [and we will add Georgia] stands almost alone in her attitude of toleration. Of one result of this state of affairs we are all clearly aware. The action of neighboring States, near and more distant, in requiring irregular practitioners to move on and to stand not upon the order of their going, has brought to our too hospitable territory a horde of medical pretenders who have not been slow in discovering the advantages of an asylum here."

By the present law, if it may be called a law, a diploma is a license. There could not be a more unfortunate blunder. The diploma-conferring power is much abused by American Medical Colleges, and the possession of a degree is by no means a guarantee of fitness to practice.

Such is the fact; what is the remedy? There is only one. If Georgia would like to have her citizens protected from the dangers of incapable and dishonest practitioners; if she would like to rid herself of those miserable blood-suckers, the despised and rejected of other States; if she would like to divorce the right to practice medicine from the empty honor of having a diploma; if she would like to bring about higher standards

*_Boston Medical and Surgical Journal_, June 9, 1892.

in her medical schools and stimulate her students to higher aims; if she would like to improve the *personnel* of the medical profession in the State and endeavor to make it what it should be, an intelligent, honest and conscientious body of physicians; then let there be established a Board of Medical Examiners who shall be untrammeled of any college connections, and who shall determine whether a given applicant is qualified to practice medicine in Georgia.

